# Low-Dose Fluvoxamine Modulates Endocytic Trafficking of SARS-CoV-2 Spike Protein: A Potential Mechanism for Anti-COVID-19 Protection by Antidepressants

**DOI:** 10.3389/fphar.2021.787261

**Published:** 2021-12-16

**Authors:** Oleg O. Glebov

**Affiliations:** ^1^ Institute of Neuroregeneration and Neurorehabilitation, Qingdao University, Qingdao, China; ^2^ Department of Old Age Psychiatry, The Institute of Psychiatry, Psychology and Neuroscience, King's College London, London, United Kingdom

**Keywords:** SARS-CoV-2, endocytosis, antidepressants, drug repurposing, COVID-19

## Abstract

Commonly prescribed antidepressants may be associated with protection against severe COVID-19. The mechanism of their action in this context, however, remains unknown. Here, I investigated the effect of an antidepressant drug fluvoxamine on membrane trafficking of the SARS-CoV-2 spike protein and its cell host receptor ACE2 in HEK293T cells. A sub-therapeutic concentration (80 nM) of fluvoxamine rapidly upregulated fluid-phase endocytosis, resulting in enhanced accumulation of the spike-ACE2 complex in enlarged early endosomes. Diversion of endosomal trafficking provides a simple cell biological mechanism consistent with the protective effect of antidepressants against COVID-19, highlighting their therapeutic and prophylactic potential.

## Introduction

A year and a half of the COVID-19 pandemic has brought about ongoing disruption of daily activities, severe economic downturn and unprecedented healthcare crisis across most of the world’s nations. Although relatively rapid COVID-19 vaccine development has been generally considered a success, vaccine availability is still limited by the logistical difficulties pertaining to production, storage and distribution (Wouters, 2021); furthermore, periodic emergence of novel strains of the SARS-CoV-2 virus fuels recurring concerns about vaccine efficacy and future-proofing (Mahase, 2021). Therefore, repurposing of cheap, readily available and safe drugs against COVID-19 remains a major healthcare priority.

One promising group of candidate drugs is antidepressants, which have been linked to protection against severe COVID-19 in a retrospective cohort study (Hoertel et al., 2021), and may block SARS-CoV-2 infection and replication in cell models (Carpinteiro et al., 2020; Eugene, 2020; Fred et al., 2021; Zimniak et al., 2021). Foremost amongst these is a generic selective serotonin reuptake inhibitor (SSRI) fluvoxamine, which has demonstrated protective potential against severe COVID-19 in small clinical studies (Lenze et al., 2020; Seftel and Boulware, 2021) and more recently in a large randomised platform trial (Reis et al., 2021). The suggested protective effect of fluvoxamine has been tentatively linked to modulation of immune response through activation of the ER sigma receptor (Hashimoto, 2021), or blockade of acid sphingomyelinase (Carpinteiro et al., 2020; Schloer et al., 2020; Corre and Loas, 2021), however, the underlying cell biological mechanism remains unknown.

## RESULTS

**FIGURE 1:**
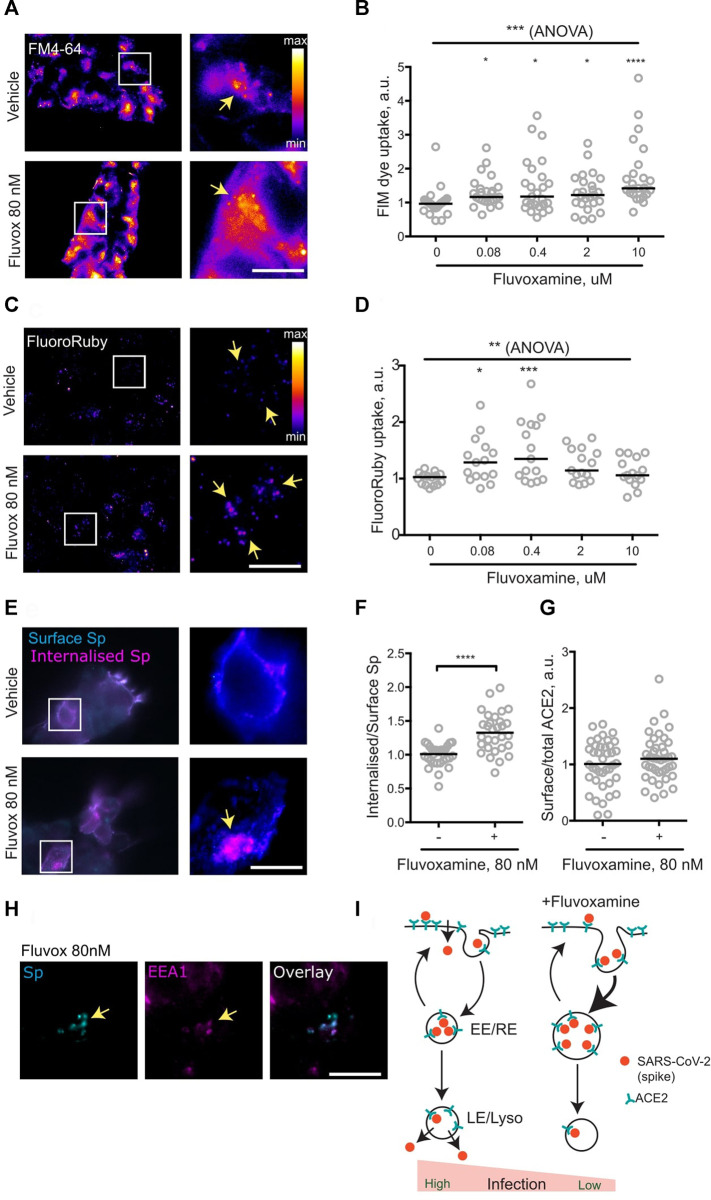
Fluvoxamine modulates endocytosis, including that of SARS-CoV-2 spike protein. **(A)**, HEK293T cells were allowed to internalise FM dye for 1 h. Representative images of cells incubated with vehicle or 80 nM fluvoxamine. Arrows denote puncta of internalised label. **(B)**, Quantification of FM dye uptake experiments for various concentrations of fluvoxamine. ***p = 0.0001, Kruskal-Wallis test. ****p < 0.0001, *p < 0.05, Dunn’s multiple comparisons test. **(C)**, HEK293T cells were allowed to internalise FluoroRuby for 1 h. Representative images of cells incubated with vehicle or 80 nM fluvoxamine. Arrows denote puncta of internalised label. **(D)**, Quantification of FluoroRuby uptake experiments for various concentrations of fluvoxamine. **p < 0.01, 1-way ANOVA; ***p < 0.001, *p < 0.05, Holm-Sidak’s multiple comparisons test. **(E)**, HEK293T cells were transfected with a plasmid expressing human ACE2, incubated with the recombinant SARS-CoV-2 spike protein for 1 h and sequentially immunostained for surface (cyan) and internalised (magenta) spike protein. Representative images of cells incubated with vehicle or 80 nM Fluvoxamine. Arrows denote apparent puncta of internalised spike protein. **(F)**, Quantification of the spike protein uptake assay. ****p < 0.0001, unpaired two-tailed *t* test. **(G)**, Quantification of surface ACE2 (live-labelled by spike protein) vs. total ACE2 labelling following 1 h treatment with 80 nM fluvoxamine. *p* > 0.05, Mann Whitney test. **(H)**, Accumulation of Spike in EEA1-positive early endosomes following 1 h treatment with vehicle or 80 nM fluvoxamine. Arrows denote apparent puncta of internalised spike protein colocalising with EEA1 puncta. **(I)**, Proposed model for modulation of SARS-CoV-2 endocytic trafficking by fluvoxamine. Fluvoxamine promotes accumulation of SARS-CoV-2 into early/recycling endosomes (EE/RE), thereby diverting it away from cell entry through the plasma membrane or late endosomes/lysosomes (LE/Lyso). Scale bar, 10 μm.

It had been suggested that pharmacological modulation of SARS-CoV-2 membrane trafficking might offer a strategy for both treatment and prophylaxis of COVID-19, with a list of candidate drugs including fluvoxamine (Glebov, 2020b). Given the emergence of clinical data suggesting efficacy of fluvoxamine against COVID-19, the effect of fluvoxamine treatment on membrane trafficking was assessed in a cell line. To this end, HEK293T cells were treated with a range of fluvoxamine concentrations. Although standard dosing of fluvoxamine results in considerable brain enrichment of the drug (10 μM) (Bolo et al., 2000), its concentrations in the blood plasma are much lower (Kasper et al., 1993), therefore a range of concentrations was used. Treatment for 1 h resulted in a significant increase in endocytosis, as measured by the membrane FM dye uptake (Figures 1A,B). Remarkably, this increase was apparent at as little as 80 nM fluvoxamine, equivalent to approximately 1/5th of the blood plasma concentration (Kasper et al., 1993).

To verify upregulation of endocytosis, uptake of the fluid-phase biologically inert endocytic marker fluorescent dextran FluoroRuby was also measured. Treatment with low concentrations of fluvoxamine resulted in a significant increase in FluoroRuby uptake mirroring the effect on the FM dye uptake, confirming the increase in both the membrane area and the volume of endocytosis (Figures 1C,D). Taken together, these observations suggest that sub-therapeutic doses of fluvoxamine may rapidly alter endocytosis in a non-specific manner.

The non-specific nature of the fluvoxamine effect suggested that it could also affect endocytic trafficking of SARS-CoV-2 as postulated previously (Glebov, 2020b). To investigate this possibility, the key steps of SARS-CoV-2 endocytosis were recreated by imaging internalisation of recombinant SARS-CoV-2 spike protein (McKay et al., 2020) into HEK293T cells transiently transfected with a plasmid expressing the human SARS-CoV-2 receptor ACE2 (Ng et al., 2020). Consistent with the effect on fluid-phase and membrane endocytosis, 1 h incubation with 80 nM fluvoxamine resulted in a significant increase in spike protein internalisation, as measured by the ratio of the internal spike labelling to that at the surface (Figures 1E,F). Relative levels of surface-bound spike protein were not changed following 1 h treatment, suggesting that steady-state surface levels of ACE2 were unaffected by the drug treatment (Figure 1G). From these results it can be concluded that fluvoxamine treatment upregulates endocytosis of spike protein.

Following endocytosis, internalised cargoes proceed to early endosomes, where they undergo sorting to be recycled back to the cell surface or trafficked into late endosomes/lysosomes; early endosomes have also been implicated in coronaviral infection (Wang et al., 2008; Khan et al., 2020; Schloer et al., 2020; Bayati et al., 2021; Shang et al., 2021). To investigate the effect of fluvoxamine treatment on early endosomes, cells were immunostained for a canonical early endosome marker EEA1. In line with previously published data (Wang et al., 2008), internalised spike protein co-localised with EEA1-positive puncta, suggesting that early/recycling endosomes are likely to be involved in SARS-CoV-2 trafficking (Figure 1H). Fluvoxamine treatment enlarged EEA1 positive puncta (Supplementary Figure S1A), consistent with increased endosomal capacity as evidenced by the increase in fluid-phase uptake; in contrast, the morphology of late endosomes as shown by immunostaining for a late endosome marker LAMP1 was not visibly affected.

Two more antidepressants identified as candidates for modulating SARS-CoV-2 membrane trafficking (Glebov, 202b) were a SSRI sertraline and a tricyclic drug imipramine, both of which have recently been shown to block SARS-CoV-2 infection in epithelial cells (Carpinteiro et al., 2020). Their effect on endocytosis was tested as described above. Treatment with low therapeutic concentrations of sertraline (80 nM) and imipramine (200 nM) resulted in an increase in endocytosis as evidenced by FM dye uptake (Supplementary Figures S1B,C). Higher concentrations of sertraline did not produce an increase in FM uptake, consistent with either cytotoxicity or previously reported inhibition of dynamin function and endocytosis blockade (Takahashi et al., 2010). Global upregulation of endocytosis may therefore represent a generalised feature of low-dose antidepressant treatment.

## DISCUSSION

To summarise, it was found that a short-duration (1 h) treatment with a sub-therapeutic concentration of fluvoxamine resulted in a acute rearrangement of membrane trafficking in a human cell line, enhancing endocytic uptake of SARS-CoV-2 spike protein into early endosomes (Figure 1I). While a recent preprint reports no effect of submicromolar concentrations of fluvoxamine and other drugs on SARS-CoV-2 infection over 24 h (Fred et al., 2021), it is possible that the actual drug concentrations were lower due to metabolism by the cells and the culture medium (Hoffmann et al., 2021). Depending on the cell type, cell entry of SARS-CoV2 may involve fusion at the cell surface or in the late endosome/lysosomes (Burkard et al., 2014; Milewska et al., 2018; Szczepanski et al., 2018; Grimm and Tang, 2020; Ghosh, 2020; Khan et al., 2020; Schloer et al., 2020), and the exact mode of SARS-CoV-2 ingress into the respiratory epithelial tissue in COVID-19 remains unclear (Murgolo et al., 2021). Regardless of the entry site, trafficking to the early/recycling endosome may provide a mechanism for rerouting SARS-CoV-2 away from cell entry, suggesting a cell biological process underlying the emerging clinical benefits of fluvoxamine.

The molecular mechanism of this effect remains to be determined. Sigma receptor-independent mode of action is supported by the similarity in the effects of low concentrations of fluvoxamine (sigma receptor agonist) and sertraline (sigma receptor antagonist). At high concentrations, many antidepressants and antipsychotics may affect lipid dynamics through inhibition of acid sphingomyelinase (Kornhuber et al., 2011) or direct manipulation of the lipid bilayer (Kapoor et al., 2019) resulting in phospholipidosis (Tummino et al., 2021) and toxicity. Nevertheless, the low fluvoxamine concentration used in this study has consistently shown no effect on cell viability in various cell types (Rafiee et al., 2016; Ghareghani et al., 2017; Tong et al., 2020) and is well below the typical threshold for phospholipidosis, suggesting the existence of an alternative process.

Only the drugs from the previously published list of candidates (Glebov, 2020b) were included in this work, with most of the focus being on fluvoxamine due to its burgeoning clinical potential. However, emerging laboratory and clinical evidence hints at the possible therapeutic potential of other antidepressants (Carpinteiro et al., 2020; Schloer et al., 2020; Fred et al., 2021; Hoertel et al., 2021; Zimniak et al., 2021). Moreover, diversion of SARS-CoV-2 away from its productive infectious route implies that antidepressants may be amenable not only for COVID-19 treatment, but also for prophylaxis. The wide prevalence of SSRIs in treatment of moderate psychiatric disorders will allow for detailed investigation of their prophylactic potential, i.e. by probing for association between SSRI prescription and COVID-19 incidence rates in the general population.

It must be emphasised that, given the ongoing proliferation of non-peer-reviewed COVID-19 publications (Brierley, 2021), the findings reported here should be interpreted with caution and chiefly considered as a basis for further investigation. Importantly, antidepressant-dependent SARS-CoV-2 membrane trafficking needs to be tracked in more detail and verified in a physiologically relevant context, ideally featuring infection of human respiratory epithelial tissue with live SARS-CoV-2 particles (Synowiec et al., 2021). Finally, the reported impact on membrane trafficking mechanisms may be of relevance in the context of multiple side-effects and drug interactions associated with antidepressants, suggesting that cell biology of psychiatric drugs outside the central nervous system (Glebov, 2020a) merits further investigation.

## Materials and Methods

### Materials

Cell culture materials were from Gibco. Drugs were from Sigma-Aldrich. FM Dyes and FluoroRuby were from Thermo Fisher. Recombinant SARS-CoV-2 spike protein and the anti-spike mouse polyclonal antibody were kindly provided by Dr. Paul F. McKay (Department of Infectious Disease, Faculty of Medicine, Imperial College London) through the COVID-19 Crowdfight initiative. The following rabbit antibodies were used: anti-EEA1, anti-ACE2 (GeneTex), anti-LAMP1 FITC conjugated (Sino Biological), anti-6xHis HRP conjugated (Abcam). Secondary antibodies were from Jackson Immunoresearch.

### Cell Culture and Uptake Assays

HEK293T cells were cultured in Dulbecco's Modified Eagle Medium (DMEM) supplemented with 10% bovine serum. For experiments, cells were grown on 13 mm round thickness 1.5 coverslips coated with poly-l-Lysine. Cells were transfected using Lipofectamine 2000 and used 24–48 h afterwards. All uptake experiments were performed at 37°C. For uptake experiments, probes were diluted in culture medium and added to the coverslips in presence of indicated concentrations of drugs or vehicle (DMSO) for indicated periods of time. FM dyes (FM4-64fx and FM1-43fx) were used at the final concentration of 20 μg/ml, FluoroRuby was used at the final concentration of 0.1% w/v, Spike protein was used at the final concentration of 0.14 μg/ml. For FM dye and FluoroRuby uptake measurements, cells were fixed, mounted and imaged without further processing.

### Immunocytochemistry and Microscopy

All steps were performed at room temperature. Cells were fixed for 10 min in 4% w/v paraformaldehyde in PBS, permeabilised in the ICC buffer (0.1% w/v Triton X-100, 5% w/v horse serum in PBS), stained with the primary antibodies for 1 h. The following antibody dilutions (v/v) were used: 1/1,000 (anti-spike), 1/1,000 (anti-6xHis), 1/500 (anti-ACE2), 1/400 (anti-EEA1), 1/400 (anti-LAMP1). For spike protein uptake measurements, cells were fixed, immunostained with the anti-6xHis antibody for 30 min to label the surface pool, permeabilised, immunostained with an anti-spike antibody for 30 min to label the total spike pool. For surface and total ACE2 measurements, coverslips were live-labelled with spike protein for 30 min before fixation to label the surface pool of ACE2. Following fixation and permeabilisation, coverslips were labelled with the anti-ACE2 antibody to visualise the total pool of ACE2. Finally, coverslips were incubated with the anti-rabbit-Alexa Fluor 488 and anti-mouse Alexa Fluor 594 secondary antibodies for 30 min. Coverslips were mounted in Fluoromount-G medium (Southern) and imaged in the EVOS M7000 widefield imaging system (Thermo Fisher) using the ×100 objective. Quantification of the images was carried out in ImageJ. For measurement of spike internalisation, intensity of total spike labelling was divided by intensity of the surface spike labelling. All data was normalised to the median values in the vehicle-treated sample.

### Statistical Analysis

Datasets represent results of at least three independent experiments. Statistical analysis was carried out using the Prism 6.0c software package (GraphPad Software). Data distributions were assessed for normality. For normally distributed datasets, Student’s t-test, 1-way ANOVA and Holm-Šidák’s post-test were used; otherwise, Mann-Whitney rank test, Kruskal-Wallis test and Dunn’s post-test were used. Datasets were presented as scatter dot plots with line at median or as cumulative probability plots.

## Data Availability

The raw data supporting the conclusion of this article will be made available by the author, without undue reservation.

## References

[B1] Bayati A. Kumar R. Francis V. McPherson P. S. (2021). SARS-CoV-2 Infects Cells after Viral Entry via Clathrin-Mediated Endocytosis. J. Biol. Chem. 296, 100306. 10.1016/j.jbc.2021.100306 33476648 PMC7816624

[B2] Bolo N. R. Hodé Y. Nédélec J. F. Lainé E. Wagner G. Macher J. P. (2000). Brain Pharmacokinetics and Tissue Distribution *In Vivo* of Fluvoxamine and Fluoxetine by Fluorine Magnetic Resonance Spectroscopy. Neuropsychopharmacology 23, 428–438. 10.1016/S0893-133X(00)00116-0 10989270

[B3] Brierley Liam (2021). Lessons from the Influx of Preprints during the Early COVID-19 Pandemic - the Lancet Planetary Health. Lancet Planet. Health 5, 3e108–e175. 10.1016/S2542-5196(21)00011-5 33713612

[B4] Burkard C. Verheije M. H. Wicht O. van Kasteren S. I. van Kuppeveld F. J. Haagmans B. L. (2014). Coronavirus Cell Entry Occurs through the Endo-/Lysosomal Pathway in a Proteolysis-dependent Manner. Plos Pathog. 10, e1004502. 10.1371/journal.ppat.1004502 25375324 PMC4223067

[B5] Carpinteiro A. Edwards M. J. Hoffmann M. Kochs G. Gripp B. Weigang S. (2020). Pharmacological Inhibition of Acid Sphingomyelinase Prevents Uptake of SARS-CoV-2 by Epithelial Cells. Cell Rep. Med. 1, 100142. 10.1016/j.xcrm.2020.100142 33163980 PMC7598530

[B6] Corre P. L. Loas G. (2021). Repurposing Functional Inhibitors of Acid Sphingomyelinase (Fiasmas): an Opportunity against SARS-CoV-2 Infection? J. Clin. Pharm. Ther. 46 (5), 1213–1219. 10.1111/jcpt.13390 33645763 PMC8014536

[B7] Eugene A. R. (2020). Fluoxetine Pharmacokinetics and Tissue Distribution Suggest a Possible Role in Reducing SARS-CoV-2 Titers. medRxiv 1217, 20248442. 10.1101/2020.12.17.20248442

[B8] Fred S. M. Kuivanen S. Ugurlu H. Casarotto P. C. Levanov L. Saksela K. (2021). Antidepressant and Antipsychotic Drugs Reduce Viral Infection by SARS-CoV-2 and Fluoxetine Show Antiviral Activity against the Novel Variants *In Vitro* . bioRxiv 0322, 436379. 10.1101/2021.03.22.436379 PMC8809408 35126106

[B9] Ghareghani M. Zibara K. Sadeghi H. Dokoohaki S. Sadeghi H. Aryanpour R. (2017). Fluvoxamine Stimulates Oligodendrogenesis of Cultured Neural Stem Cells and Attenuates Inflammation and Demyelination in an Animal Model of Multiple Sclerosis. Sci. Rep. 7, 4923. 10.1038/s41598-017-04968-z 28687730 PMC5501834

[B10] Glebov O. O. (2020a). Tonic NMDA Receptor Signalling Shapes Endosomal Organisation in Mammalian Cells. Sci. Rep. 10, 9315. 10.1038/s41598-020-66071-0 32518335 PMC7283358

[B11] Glebov O. O. (2020b). Understanding SARS-CoV-2 Endocytosis for COVID-19 Drug Repurposing. FEBS J. 287, 3664–3671. 10.1111/febs.15369 32428379 PMC7276759

[B12] Ghosh Sourish β-Coronaviruses Use Lysosomes for Egress Instead of the Biosynthetic Secretory Pathway. Cell 2020 183(6):1520-1535. 10.1016/j.cell.2020.10.039 33157038 PMC7590812

[B13] Grimm C. Tang R. (2020). Could an Endo-Lysosomal Ion Channel Be the Achilles Heel of SARS-CoV2? Cell Calcium 88, 102212. 10.1016/j.ceca.2020.102212 32402856 PMC7201244

[B14] Hashimoto K. (2021). Repurposing of CNS Drugs to Treat COVID-19 Infection: Targeting the Sigma-1 Receptor. Eur. Arch. Psychiatry Clin. Neurosci. 271, 249–258. 10.1007/s00406-020-01231-x 33403480 PMC7785036

[B15] Hoertel N. Sánchez-Rico M. Vernet R. Beeker N. Jannot A.-S. Neuraz A. (2021). Association between Antidepressant Use and Reduced Risk of Intubation or Death in Hospitalized Patients with COVID-19: Results from an Observational Study. Mol. Psychiatry 26, 5199–5212. 10.1038/s41380-021-01021-4 33536545

[B16] Hoffmann M. Hofmann-Winkler H. Smith J. C. Krüger N. Arora P. Sørensen L. K. (2021). Camostat Mesylate Inhibits SARS-CoV-2 Activation by TMPRSS2-Related Proteases and its Metabolite GBPA Exerts Antiviral Activity. EBioMedicine 65, 103255. 10.1016/j.ebiom.2021.103255 33676899 PMC7930809

[B17] Kapoor R. Peyear T. A. Koeppe R. E. Andersen O. S. (2019). Antidepressants Are Modifiers of Lipid Bilayer Properties. J. Gen. Physiol. 151, 342–356. 10.1085/jgp.201812263 30796095 PMC6400527

[B18] Kasper S. Dötsch M. Kick H. Vieira A. Möller H. J. (1993). Plasma Concentrations of Fluvoxamine and Maprotiline in Major Depression: Implications on Therapeutic Efficacy and Side Effects. Eur. Neuropsychopharmacol. 3, 13–21. 10.1016/0924-977x(93)90290-3 8471827

[B19] Khan N. Chen X. Geiger J. D. (2020). Role of Endolysosomes in Severe Acute Respiratory Syndrome Coronavirus-2 Infection and Coronavirus Disease 2019 Pathogenesis: Implications for Potential Treatments. Front. Pharmacol. 11, 595888. 10.3389/fphar.2020.595888 33324224 PMC7723437

[B20] Kornhuber J. Muehlbacher M. Trapp S. Pechmann S. Friedl A. Reichel M. (2011). Identification of Novel Functional Inhibitors of Acid Sphingomyelinase. PLOS ONE 6, e23852. 10.1371/journal.pone.0023852 21909365 PMC3166082

[B21] Lenze E. J. Mattar C. Zorumski C. F. Stevens A. Schweiger J. Nicol G. E. (2020). Fluvoxamine vs Placebo and Clinical Deterioration in Outpatients with Symptomatic COVID-19: A Randomized Clinical Trial. JAMA 324, 2292–2300. 10.1001/jama.2020.22760 33180097 PMC7662481

[B22] Mahase E. (2021). Covid-19: Where Are We on Vaccines and Variants? BMJ 372, n597. 10.1136/bmj.n597 33653708

[B23] McKay P. F. Hu K. Blakney A. K. Samnuan K. Brown J. C. Penn R. (2020). Self-amplifying RNA SARS-CoV-2 Lipid Nanoparticle Vaccine Candidate Induces High Neutralizing Antibody Titers in Mice. Nat. Commun. 11, 3523. 10.1038/s41467-020-17409-9 32647131 PMC7347890

[B24] Milewska A. Nowak P. Owczarek K. Szczepanski A. Zarebski M. Hoang A. (2018). Entry of Human Coronavirus NL63 into the Cell. J. Virol. 92. 10.1128/JVI.01933-17 PMC5774871 29142129

[B25] Murgolo N. Therien A. G. Howell B. Klein D. Koeplinger K. Lieberman L. A. (2021). SARS-CoV-2 Tropism, Entry, Replication, and Propagation: Considerations for Drug Discovery and Development. PLOS Pathog. 17, e1009225. 10.1371/journal.ppat.1009225 33596266 PMC7888651

[B26] Ng K. W. Faulkner N. Cornish G. H. Rosa A. Harvey R. Hussain S. (2020). Preexisting and De Novo Humoral Immunity to SARS-CoV-2 in Humans. Science 370, 1339–1343. 10.1126/science.abe1107 33159009 PMC7857411

[B27] Rafiee L. Hajhashemi V. Javanmard S. H. (2016). Fluvoxamine Inhibits Some Inflammatory Genes Expression in LPS/stimulated Human Endothelial Cells, U937 Macrophages, and Carrageenan-Induced Paw Edema in Rat. Iran. J. Basic Med. Sci. 19, 977–984. 27803785 PMC5080428

[B28] Reis G. Thabane Lehana. Milagres Aline. Cruz. Ferreira Thiago. Santiago. dos Santos Castilho. Vitor. Quirino. Ribeiro Luciene. Barra. (2021). Effect of Early Treatment with Fluvoxamine on Risk of Emergency Care and Hospitalisation Among Patients with COVID-19: the TOGETHER Randomised, Platform Clinical Trial. Lancet Glob. Health online ahead of print. 10.1016/s2214-109x(21)00448-4 PMC8550952 34717820

[B29] Schloer S. Brunotte L. Goretzko J. Mecate-Zambrano A. Korthals N. Gerke V. (2020). Targeting the Endolysosomal Host-SARS-CoV-2 Interface by Clinically Licensed Functional Inhibitors of Acid Sphingomyelinase (FIASMA) Including the Antidepressant Fluoxetine. Emerg. Microbes Infect. 9, 2245–2255. 10.1080/22221751.2020.1829082 32975484 PMC7594754

[B30] Seftel D. Boulware D. R. (2021). Prospective Cohort of Fluvoxamine for Early Treatment of Coronavirus Disease 19. Open Forum Infect. Dis. 8, ofab050. 10.1093/ofid/ofab050 33623808 PMC7888564

[B31] Shang C. Zhuang X. Zhang H. Li Y. Zhu Y. Lu J. (2021). Inhibitors of Endosomal Acidification Suppress SARS-CoV-2 Replication and Relieve Viral Pneumonia in hACE2 Transgenic Mice. Virol. J. 18, 46. 10.1186/s12985-021-01515-1 33639976 PMC7914043

[B32] Synowiec A. Szczepański A. Barreto-Duran E. Lie L. K. Pyrc K. (2021). Severe Acute Respiratory Syndrome Coronavirus 2 (SARS-CoV-2): a Systemic Infection. Clin. Microbiol. Rev. 34. 10.1128/CMR.00133-20 PMC7849242 33441314

[B33] Szczepanski A. Owczarek K. Milewska A. Baster Z. Rajfur Z. Mitchell J. A. (2018). Canine Respiratory Coronavirus Employs Caveolin-1-Mediated Pathway for Internalization to HRT-18G Cells. Vet. Res. 49, 55. 10.1186/s13567-018-0551-9 29970183 PMC6029178

[B34] Takahashi K. Miyoshi H. Otomo M. Osada K. Yamaguchi N. Nakashima H. (2010). Suppression of Dynamin GTPase Activity by Sertraline Leads to Inhibition of Dynamin-dependent Endocytosis. Biochem. Biophys. Res. Commun. 391, 382–387. 10.1016/j.bbrc.2009.11.067 19913505

[B35] Tong X. Y. Liao X. Gao M. Lv B. M. Chen X. H. Chu X. Y. (2020). Identification of NUDT5 Inhibitors from Approved Drugs. Front. Mol. Biosci. 7, 44. 10.3389/fmolb.2020.00044 32300600 PMC7145388

[B36] Tummino T. A. Rezelj V. V. Fischer B. Fischer A. O'Meara M. J. Monel B. (2021). Drug-induced Phospholipidosis Confounds Drug Repurposing for SARS-CoV-2. Science 373, 541–547. 10.1126/science.abi4708 34326236 PMC8501941

[B37] Wang H. Yang P. Liu K. Guo F. Zhang Y. Zhang G. (2008). SARS Coronavirus Entry into Host Cells through a Novel Clathrin- and Caveolae-independent Endocytic Pathway. Cell Res 18, 290–301. 10.1038/cr.2008.15 18227861 PMC7091891

[B38] Wouters Olvier. J. (2021). Challenges in Ensuring Global Access to COVID-19 Vaccines: Production, Affordability, Allocation, and Deployment. The Lancet 397, 1023–1034. 10.1016/S0140-6736(21)00306-8 PMC7906643 33587887

[B39] Zimniak M. Kirschner L. Hilpert H. Geiger N. Danov O. Oberwinkler H. (2021). The Serotonin Reuptake Inhibitor Fluoxetine Inhibits SARS-CoV-2 in Human Lung Tissue. Sci. Rep. 11, 5890. 10.1038/s41598-021-85049-0 33723270 PMC7961020

