# Supplementary material for: Low-Dose Fluvoxamine Modulates Endocytic Trafficking of SARS-CoV-2 Spike Protein: A Potential Mechanism for Anti-COVID-19 Protection by Antidepressants
Source: Front Pharmacol. 2021 Dec 16;12:787261. doi: 10.3389/fphar.2021.787261 (PMC8716620; doi:10.3389/fphar.2021.787261)
Supplement: Supplementary file 1 [file Image1.pdf]

Figure S1

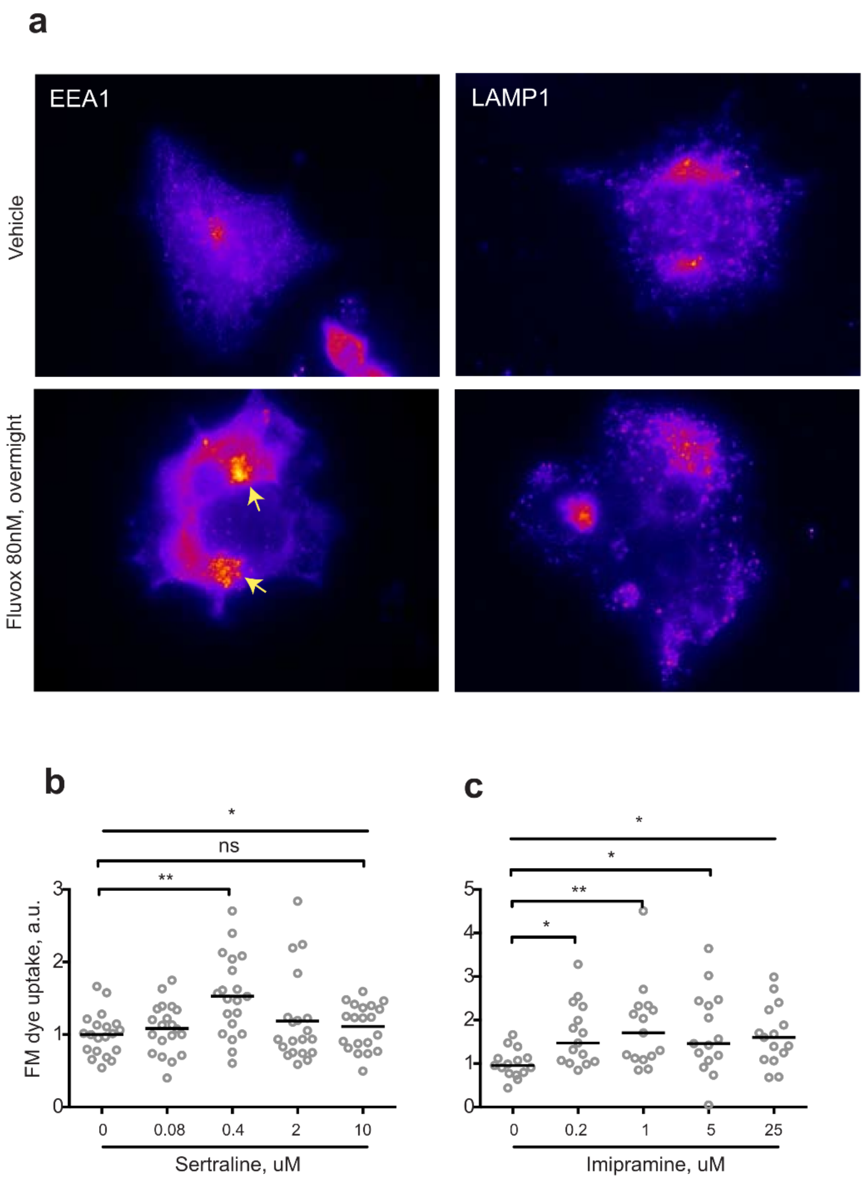

Figure S1. Supplementary data. a, HEK293T cells were treated with vehicle or 80 nM fluvoxamine overnight and immunostained for EEA1 or LAMP1. Arrows denote enlarged early endosomes following fluvoxamine treatment. **b**, Quantification of FM dye uptake experiments for various concentrations of sertraline. *P<0.05, Kruskal-Wallis test. **P<0.01, Dunn's multiple comparisons test. **c**, Quantification of FM dye uptake experiments for various concentrations of imipramine. *P<0.05, Kruskal-Wallis test. *P<0.05, **P<0.01, Dunn's multiple comparisons test. Scale bar, 20 um
